# Treatment of Cancer in the Older Aged Person.

**DOI:** 10.4084/MJHID.2010.029

**Published:** 2010-09-29

**Authors:** Lodovico Balducci

## Abstract

Cancer is a disease of aging.^1^ Currently 50% of all malignancies occur in individuals 65 and over^1^ and by the year 2030 older individuals will account for 70% of all neoplasms.

With the aging of the population the management of cancer in the older person with chemotherapy is beoming increasingly common. This treatment may be safe and effective if some appropriate measures are taken, including, an assessment of the physiologic age of each patient, modification of doses according to the renal function, use of meyelopoietic growth factors prophylactically in presence of moderately toxic chemotherapy, and provision of an adequate caregiver. Cure, prolongation of survival, and symptom palliation are universal goals of medical treatment. Prolongation of active life expectancy should be added to the treatment goal of the older aged person.

## Introduction:

The management of cancer in the older aged person is an increasingly common problem. Cancer is a disease of aging.^1^ Currently 50% of all malignancies occur in individuals 65 and over^1^ and by the year 2030 older individuals will account for 70% of all neoplasms.

The management of cancer in the older-aged person include some questions that are specfic of aging:

- Is the person life expectancy going to be shortened by cancer?
- Is the patient’s life expectancy long enough that he or she will experience the complications of cancer?
- Is the patient able to tolerate antineoplastic treatment?
- What are the long term side effects of cancer treatment in the older person?
- Does the patient have adequate social support to undergo cancer treatment?

We will address these questions using cytotoxic chemotherapy as a model. After an overview of aging and its assessment we will explore the pharmacologic changes of aging and the provisions to ameliorate the complications of chemotherapy.

## Age and its assessment:

Aging implies a decline in life expectancy and stress-coping ability, increased prevalence of comorbidity, increased risk of functional dependence and of the need of social support.^2^ Though it is universal, aging is highly individualized and is poorly reflected in chronologic age. The management of the older aged person should be based on an assessment of physiologic rather than chronologic age.

Aging has been defined as loss of entropy and fractality^3^ and as loss of homeostasis.^4^ Entropy reflects the ability of a system to produce and to waste energy. A fractal is a unit subdividing into subunits of the same type, but whose number and size are unpredictable, similar to the branch of a tree. The maintenance of life is trusted to structural and dinamic fractals. In the mammalian body structural fractals include the respiratory, circulatory and nervous systems; the dinamic fractals include the process of cell generation. For example, in hemopoiesis, few pluripotent stem cells give origin to a number of committed progenitors from which the differentiated precursors of the circulating blood cells are derived. Commitment and differentiation involve the branching of the pluripotent stem cell into progressively larger cell populations. Entropy and fractality cannot be directly assessed by clinical means. The loss of cell replicating ability, expressed by a reduced length of leukocyte telomeres,^5^ may be seen as an expression of the loss of fractality. A number of studies have explored the possibility that the length of leukocyte telomeres provide a reliable assessment of physiologic age, but correlating the length of telomeres to the risk of functional dependence, geriatric syndromes, and other manifestations of age. The results so far have been inconclusive.^5^–^10^

Homeostasis is the ability of a system to restore basic conditions after stress imposed by environmental interactions. One may observe the dysregulation of a number of physiologic parameters, including blood pressure, insulin sensitivity, circulating levels of corticosteroid and cathecolamines. The so called “allostatic load” assesses the dysregulation of 12 different parameters and may estimate the physiologic age. Its clinical value so faris unestablished.^4^ Chronic and progressive inflammation is arguably the best recognized manifestation of allostasis.. Aging is associated with increased concentration of circulating inflammatory markers, including inflammatory cytokines and fibrinolytic products. The concentration of Interleuking 6, D-Dimer, and C-reactive protein is associated with increased risk of death, functional dependence and geriatric syndromes^11^–^14^ and may mirror the physiologic age of the person.

Inflammatory markers and the length of leukocyte telomeres are promising laboratory tests, but at present have limited clinical use.

The best validated instrument for the assessment of chronologic age is the Comprehensive Geriatric Assessment” (CGA), whose elements are illustrated in **Table 1**.^15^–^17^

Dependence in one or more ADLs and the presence of one or more geriatric syndromes imply a marginal functional reserve associated with very limited tolerance of stress. These individuals can only survive thanks to a full time home caregiver, or to admission to an adult living facility. Dependence in one or more IADLs is associated increased risk of mortality,^18^–^21^ of dementia, and of chemotherapy-induced toxicity.^20^–^21^ These individuals need a caregiver to negotiate the outside world.

In addition to reduced life-expectancy and increased risk of treatment complications^22^–^24^ comorbidity may be associated to changes in the natural history of cancer and polypharmacy. It is usefule to mention that diabetes has been associated with worsened prognosis of most common neoplasms including cancer of the large bowel, of the prostate and of the breast.^22^,^25^ Anemia deserves a special mention as it is associated with increased risk of overall mortality in older individuals, of functional dependence and of chemotherapy-induced myelotoxicity.^26^ Polypharmacy may be responsible of iatrogenic morbidity and of unfavorable interactions with antineoplastic drugs.^27^–^28^ In addition, drug interactions are a major cause of iatrogenic morbidity.

Some elements of the CGA may be utilized in models that predict the mortality and the risk of chemotherapy-related toxicity in older individuals.^20^–^21^,^29^–^31^ In addition, the CGA identify reversible conditions that interfere with cancer treatment, including comorbidity, malnutrition, absence of a reliable caregiver.^32^–^34^

Any discussion of the assessment of physiologic age should include frailty. This is constructed as a condition of critically reduced physiologic reserve so that a minimal stress may cause loss of independence and start a chain of events that lead to the patient ‘s death.^3^

The clinical definition of frailty was first provided by the Cardiovascular Health Study (CHS).^35^ Eighty-five hundred individuals 65 and over were followed for an average of 11 years. Based on five simple parameters they could be divided into three groups with different risk of mortality, disability, and admission to adult living facilities. The parameters of interest included:

- Involuntary weight loss of 10 lbs or more over a 6 months period;
- Decreased grip strength;
- Difficulty in starting movements;
- Reduced walk speed;
- Exhaustion.

The three groups of individuals were classified as: non-frail or fit (no abnormalities); pre-frail (up to two abnormalities), and frail (three or more abnormalities).

Another index of frailty validated both in older women and older men has been provided by the Study of Osteoporotic Fracture (SOF)^36^ and older men.^37^ Based on the performance of simple exercises (rising five times from a chair) it appears as accurate a predictor of falls, death, fractures and disability as the CHS instrument. Canadian investigators developed a frailty index^38^ involving the accumulation of 40 functional deficits. This appears too time-consuming for clinical practice. The CHS classification represents the golden standard for ongoing studies of frailty. Fatigue was the first harbinger of frailty in about 73% of the cases detected in the CHS.^39^ This finding is germane to our discussion as fatigue is the most common chronic manifestation of cancer.

The interactions of aging and frailty should be clarified. In particular we should ask: is cancer a cause of frailty? Is chemotherapy a cause of frailty? Is frailty associated with increased risk of therapeutic complications?

## Cytotoxic chemotherapy:

Aging is associated with pharmacokinetic and pharmacodinamic changes that may enhance the toxicity of these agents (**Table 2**).^40^

The most common pharmacokinetic changes of age include reduced glomerular filtration rate, which prevents renal excretion of drugs and their metabolites, and reduced vD of hydrosoluble drugs, that is associated with increased AUC of these agents. The Vd is influences by body composition, serum albumin and hemoglobin concentration. Of these only the hemoglobin concentration can be modified at least in a short term. As discussed previously, anemia, is associated with increased risk of chemotherapy-related toxicity because several medications are bound to red blood cells. In presence of anemia the concentration of fre drugs is increased.

It is not clear at present whether an age-associated decline in food absorption affects the bio-availability of oral drugs. This issue need to be studies

Both the splanchnic circulation and the hepatic mass decrease with age. These changes conjure to reduce drug metabolism, especially when the cytochrome P450 reactions are involved. In addition, polypharmacy may modulate the cytochrome P450 system and inhibit or accelerate the metabolism of a number of drugs. The biliary excretion appears unaffected by age, but it is important to remember that some drugs, including idarubicin, daunorubicin, and morphine, give origine to active and toxic metaboites eliminated by the kidneys. The toxicity of these agents may then be enhanced in the presencce of declining GFR.

Some complications of cytotoxic chemotherapy become more common with aging.

The risk of neutropenia, neutropenic infections, and infectious deaths increase with the age of the patient.^41^ Fortunately, the Granulocyte Colony Stimualting Factors (G-CSF) filgrastim, pegfilgrastim and lenograstim are effective in older individuals and reduce by more than 50% the risk of infections.^42^–^43^ The use of these compounds has been associated with increased risk of myelodysplasia and acute myeloid leukemia in older individuals.^44^ The risk of thrombocytopenia and anemia also increase with age, albeit to a lesser extent. These complications are rarely lethal but may cose a reduction of the dose intensity of chemotherapy and compromise the control of the tumor. Erythopoietic stimulating agents reverse the majority of chemotherapy-related anemias, but the use of these compounds is currently limited out of concern for hypertension, thrombosis and enhanced tumor growth.^45^

Mucositis, whose incidence and severity increase with age, causes dysphagia, diarrhea and rapid volume depletion in older individuals. Aggressive fluid resuscitation should be initiated in patients unable to ingest fluids. The prevention of mucositis has limited effectiveness and may include solutions of glutamine, supersaturated solutions of calcium phosphate, and keratynocyte growth factor.^4^ Fatigue is a feeling of exhaustion not releived by rest and is the most common long term complication of cancer chemotherapy.^47^–^48^ The pathogenesis of chemotherapy induced fatigue is poorly understood, Correction of anemia may improve fatigue in some patients. In older individuals, fatigue is a a harbinger of frailty.^39^

Age is also a risk factor for anthracycline cardiomyopathy and for peripheral neuropathy.^40^ The risk of cardiomyopathy may be reduced by the administration of doxorubicin as a continuous infusion, by the concomitant administration of doxorubicin and the antidote desrazoxane, or by the use of pegylated liposomal doxorubicin “in lieu” of doxorubicin. None of these approaches is routinely used in older individuals, because of cost, alternative toxicity and also because the dose of doxorubicin employed is rarely associated with cardiotoxic complications.

No antidote for peripheral neurotoxiciy exists. This complication may impair the independence of older individuals by impeding both walking and fine hand movements. The only prevention consists in trying to avoid combination of neurotoxic drugs (example cisplatin and paclitaxel) and in interrupting treatment when the neuropathy may interfere with an individual’s activity.

An important and unanswered question is whether age is a risk factor for more frequent and more severe manifestations of “chemobrain” that is a cognitive dysfunction caused by chemotherapy.

Age is a risk factor for delayed complications of chemotherapy. Myelodysplasia and acute myeloid leukemia may develop in 1–2% of patients 65 and over who had received anthracycline-containing treatment in the previous 10 years. The risk of these complications is enhanced by myelopoietic growth factors.^44^ The incidence of a chronic cardiomyopathy, manifested by a progressive decline of the ejection fraction, is seen in approximately 19% of individuals 65 an older 5 years or longer after treatment with an anthracycline.^49^

Other potential long term complications of cancer chemotherapy include dementia, functional dependence and frailty.

## Treatment goals:

Cure, prolongation of survival, and symptom palliation are universal goals of medical treatment. Prolongation of active life expectancy should be added to the treatment goal of the older aged person.

The construct of active life expectancy^50^ include preservation of functional independence (that is ability to carry on ADLs and IADLs) as well as preservation of the ability to perform activities that are pleasearuble and fulfilling(the so-called advanced activities of daily living). For this purpose it is important to obtain a so called” value history” at the beginning of treatment to detect what are the patients main objectives in the lasting years of his/her life and to make sure that the treatment is aimed to preserve these objectives.

## Conclusions:

With the aging of the population the management of cancer in the older person with chemotherapy is beoming increasingly common. This treatment may be safe and effective if some appropriate measures are taken, including, an assessment of the physiologic age of each patient, modification of doses according to the renal function, use of meyelopoietic growth factors prophylactically in presence of moderately toxic chemotherapy, and provision of an adequate caregiver. The goals of treatment should include prolongation of active life-expectancy.

## Figures and Tables

**Table 1:** Comprehensive Geriatric Assessment (CGA)

**Domain**	**Assessment**

**Function**	**Performance Status (PS)** **Activities of Daily Living (ADL)** Continence, transferring, grooming, dressing, feeding, utilization of the bathroom. **Instrumental Activities of daily living (IADL)** Use of transportation, ability to take medications, to use the telephone, to go shopping, to take care of finances, to provide to one’s meals **Advanced activities of daily living.** These include all activities that make the patient’s life enjoyable.
**Comorbidity**	**Number of comorbid conditions** **Comorbidity scales (assessing the seriousness of each condition)**
**Presence of Geriatric Syndromes**	**Dementia (cognitive evaluation)** **Depression (screening tests, such as the geriatric depression scale)** **Delirium** **Falls (risk of falls assessment)** **Vertigo** **Spontaneous fractures** **Failure to thrive (weigh loss in face of normal food intake)** **Neglect and abuse**
**Social support**	
	**Living conditions** **Presence and reliability of the caregiver** **Economical resources**
**Nutrition**	**Nutritional status** **Nutritional risk (typical meals, access to food, etc)**
**Polypharmacy**	**Number of medications** **Risk of drug interactions**

**Table 2.** A pharmacokinetic changes of aging

**Parameter**	**Age-related change**
Absorption and bioavailability	Probably decreased
Volume of distribution (Vd)	Decreased for water-soluble agents ---→ increased plasmatic concentration Increased for lipid-soluble agens
Metabolism	Decreased hepatic metabolism
Excretion	Renal: decreased Biliary: not affected

**Table 3.** Common complications of cytotoxic chemotherapy

**Universal complications**	**Complications specific of certain agents**
• Myelosuppression	• Cardiotoxicity: anthracyclines
• Nausea and vomiting	• Neurotoxicity (peripheral): alkaloids, taxanes, epothlilones, platinum derivatives
• Mucositis	• Neurotoxicity (central): cytarabin in high doses, methotrexate in high doses; ifosphamide; gemcitabine
• Fatigue	• Pulmonary fibrosis: bleomycin, methotrexate
	• Renal insufficiency: cisplatin
